# Editorial: Sharing One’s Experience and Advancing Medicine through Case Reports

**DOI:** 10.3389/fsurg.2022.906371

**Published:** 2022-06-06

**Authors:** Philipp Taussky

**Affiliations:** Department of Neurosurgery, Clinical Neurosciences Center, The University of Utah, Salt Lake City, UT, United States

**Keywords:** case reports, surgery, complications, knowledge acquisition, technique, radiology

**Editorial on the Research Topic**

***Sharing One's Experience and Advancing Medicine through Case Reports***

One of our residents recently pointed out that there are three distinct ways to learn as a surgeon: From one’s own mistakes, which is the least attractive way, from mistakes reported in the literature, and from directly witnessing other people’s mistakes. The last two instances may be the most advantageous way of learning in a surgical setting. The publication of case reports exactly addresses these last two scenarios. It allows surgeons to share their experience, insights, or mistakes with a wider audience and to create the opportunity for others to learn without making the same mistake or unknowingly facing the same challenge. In its most fundamental sense, case reports speak to a higher academic calling. They transform one’s own experience into something much larger, by publishing and sharing it, it becomes part of our common literature of collectively gained knowledge in an academic framework.

In many ways, this aspect of case reports speaks directly to the core mission of Frontiers in Surgery: To create a discussion and knowledge platform of advances and research findings in surgical practice today to continuously improve clinical management of patients and foster innovation in this field. But case reports also have limitations. In the publishing world, they are not popular, since they are rarely cited and therefore tend not to improve a journal’s impact factor. For researchers and physicians, case reports are considered minor intellectual achievements relegated to a different category in an academic CV, separate from your more impactful publications.

Yet, despite their relatively small academic stature, case reports play an important part in medicine and its long history. Case reports have been identified in an Egyptian antiquity papyrus from the 16th to 17th dynasty, circa 1,600 BC (1). Interestingly, the case reports (a few dozen have been found) follow a modern presentation: Diagnosis in the title, examination and prognosis, and treatment. Case reports are also found in Greek medical literature, in the Hippocratic Corpus, probably written around 400 BC (2). In the middle ages, case reports were disseminated in the Islamic medical literature (3). Rhazes (865–929 AD), a famous Persian physician, scholar, and philosopher, whose full name was Abu Bakr Muhamed Ibn Zakariya al-Razi, left a large collection of case reports (3). While medicine lacked major progress in Europe during the Middle Ages, during the Renaissance and Enlightenment, the study of human anatomy and physiology gradually advanced and a recent linguistic analysis of publications in the Edinburgh Medical Journal (the oldest continuing medical journal in English) from 1735 to 1985 found that by the end of the 18th century case narratives had become standardized in their structure and form (4).

The significant medical value of case reports is beautifully illustrated in this current collection of case reports published in Frontiers in Surgery, the Neurosurgery section, in 2021. Case reports, such as these presented here, transform an individual clinical experience or insight into something larger: A contribution to the literature shared with other practitioners thus advancing our knowledge and the field in a fundamental academic way by being transparent and sharing knowledge.

Some of the case reports in our collection share surgical and technical nuances, such as novel bypass surgical approaches, a novel technique for cranioplasties, the novel use of an exoscope for an orbital cavernous hemangioma resection, a rare anterior inferior cerebellar artery aneurysm treated by re-anastomosis, and the use of a novel shunt for hydrocephalus (5–9). Unusual pathologies are reported on colliding pituitary adenoma and primary pituitary lymphoma, a de-novo mutation in an infant with Pallister-Hall syndrome and radiological and clinical findings of multiple cerebellar liponeurocytomas, and an unusual case of creeping growth in a lymphoplasmacyte-rich meningioma (10–13). And finally, lessons learned from unusual complications are shared in a rare case of fourth ventricle to spinal subarachnoid space shunt migration and contrast-induced encephalopathy after endovascular treatment of an intracranial aneurysm (14, 15).

These case reports represent the best of academic medicine but also the best of what an open-access journal like Frontiers has to offer: An online platform of international researchers sharing their rare insights, surgical techniques but also surgical complications, and lessons learned. The 2021 pandemic year was difficult for all of us, but the current collection of neurosurgical case reports also illustrates that despite difficulties stretching the capabilities of our healthcare systems, surgeons worldwide continued to treat patients with neurological disease, sharing their respective experiences by contributing to the field in a fundamental academic way.
